# Treatment of Chronic Liver Fibrosis: Adipose and Bone Marrow Mesenchymal Stem Cells

**DOI:** 10.3390/ijms27125340

**Published:** 2026-06-13

**Authors:** Murat Shagidulin, Artem Venediktov, Alexei Grigoriev, Mila Ibragimova, Artur Aktemirov, Aglaya Arzhanova, Pavel Fadeev, Valekh Ashyrov, Viktoria Gartseva, Anastasia Kostysheva, Ivan Lychagin, Anna Ponomareva, Lidia Salomatina, Alina Vaniukova, Alla Nikolskaya, Sergei Pershikov, Egor Kuzmin, Ksenia Pokidova, Nikolai Zharov, Natalia Kartashkina, Yulia Basok, Nina Onishchenko, Gennadii Piavchenko, Sergei Gautier

**Affiliations:** 1 Shumakov National Medical Research Center of Transplantology and Artificial Organs, Moscow 123182, Russia; bear-38@yandex.ru (A.G.); ponomareva@gmail.com (A.P.); liansa@mail.ru (L.S.); allanik64@yandex.ru (A.N.); bjb2005@mail.ru (Y.B.); nina.a.onishchenko@mail.ru (N.O.); gautier@list.ru (S.G.); 2 Department of Transplantology and Artificial Organs, Sechenov First Moscow State Medical University, Moscow 119991, Russia; mila.ibragimova.02@mail.ru (M.I.); anastasikost@gmail.com (A.K.); ilichlis001@gmail.com (I.L.); algernon1604@gmail.com (A.V.); 3 Department of Human Anatomy and Histology, Sechenov First Moscow State Medical University, Moscow 119991, Russia; venediktov_a_a@staff.sechenov.ru (A.V.); aglayaarzhanova@gmail.com (A.A.); pavel_fadeev_02@mail.ru (P.F.); ashyrov04@mail.ru (V.A.); vikagarceva@gmail.com (V.G.); pershikov_s_v@staff.sechenov.ru (S.P.); kuzmin_e_a_1@staff.sechenov.ru (E.K.); pokidova_k_s@staff.sechenov.ru (K.P.); zharov_n_a@staff.sechenov.ru (N.Z.); kartashkina_n_l@staff.sechenov.ru (N.K.); 4 Soft Matter and Physics of Fluids Center, Bauman Moscow State Technical University, Moscow 105005, Russia; arturaktemirov@gmail.com

**Keywords:** MSC, BMSCs, AdMSCs, tissue homeostasis, niche, liver regeneration, comparative study

## Abstract

Liver fibrosis is a severe but common disease without an easy-to-access option for efficient treatment. Mesenchymal stem cells (MSCs) of different origins have been tested for antifibrotic effects in vitro, in vivo, and in clinical studies over the two last decades, although the comparative efficiency of different subtypes remains not fully understood, especially for long-term survival. In this study, we aimed to compare the long-time persistence of favorable effects in male Wistar rats with liver fibrosis treated using MSCs derived from white adipose tissue (AdMSCs) and bone marrow (BMSCs). Liver fibrosis was induced by carbon tetrachloride. We studied the survival rate; oxidative index, assessed via laser Doppler flowmetry; hepatic markers in blood plasma—albumin, alanine aminotransferase, aspartate aminotransferase, and alkaline phosphatase; the ratio of liver to body mass; histological parameters—the number of adipocytes, lymphocytes, siderophages, and Ki67^+^ cells; and the relative areas of connective tissue proper and reticular fibers. Extra mortality was only typical for fibrotic animals subjected to the sham treatment in the first two weeks. Up to Day 270 of this study, both MSC-treated groups showed barely any differences from animals undergoing the sham treatment in terms of the oxidative index and blood markers, although AdMSC-treated rats presented a more favorable histological pattern than BMSC-treated ones, considering the relative area of reticular fibers and the Ki67 cell count. This study suggests that AdMSC treatments may be more appropriate than BMSC treatments in animal liver fibrosis models, with the results showing better potential for liver tissue regeneration 9 months after treatment.

## 1. Introduction

Chronic liver diseases affect approximately 1.5 billion people worldwide, and the incidence of liver cirrhosis and fibrosis is constantly increasing [1]. Moreover, liver fibrosis is barely reversible, with only a few appropriate methods existing for its treatment, while the most efficient method, liver transplantation, is highly limited [2]. A promising treatment option comprises the usage of mesenchymal stem cells (MSCs). However, obtaining MSCs can be complicated by both medical and ethical issues. In addition, the effectiveness and performance of MSC-based strategies have been proven mainly in in vitro and in vivo studies, whereas the success of MSC-based treatments in clinical studies remains ambiguous, unveiling the importance of differentiating between MSC subtypes and investigating their efficiency in the treatment of liver fibrosis [3,4].

This difference between MSCs of various origins (bone marrow, dental pulp, foreskin, umbilical cord, etc.) can be colossal as their functions are multifaceted. For instance, MSCs are responsible for cell migration or homing, tissue repair and regeneration, immunomodulation, anti-inflammatory effects, anti-apoptotic activity, neoangiogenesis, activation of resident stem cells, and even antimicrobial effects, and this is via not only direct interaction but also due to paracrine effects, including those that are mediated by the secretion of exosomes and microvesicles [5]. While many of these mechanisms are well-studied, comprehensive comparative studies between MSC subtypes are still relevant.

For example, it has been reported that MSCs originating from the umbilical cord showed the most prominent adhesive and pro-regenerating potential, with bone marrow stem cells (BMSCs) being the second ones [6], although the application of umbilical MSCs for clinical studies is limited due to ethical reasons. Meanwhile, adipose tissue MSCs (AdMSCs) are probably the easiest to access via simple liposuction as their niche in white adipose tissue is common or even abundant in many individuals [7]. However, it is challenging to know whether AdMSCs exhibit a higher efficiency level than BMSCs, especially considering the usage of MSCs in liver fibrosis.

Basically, AdMSCs can be identified by a remarkable expression of CD105 and CD44, whereas CD45 and CD34 are almost not expressed [8]. Standard markers for AdMSCs, however, are the same as for BMSCs and also include positive CD73 and negative CD11b, CD19, and HLA-DR [9]. AdMSCs have already demonstrated their benefits for treatment of liver damage in rodent models [8,10]. There also exists a large body of evidence that AdMSCs can be as efficient as BMSCs [11].

For example, a recent study by Yoshida and colleagues in renal fibrosis reported that the choice between AdMSCs and BMSCs can be challenging [12]. On the one hand, AdMSCs were preferrable, but, on the other hand, they were associated with adverse thrombogenic events, so the authors concluded the choice between AdMSCs and BMSCs should be based on the target pathology [12]. Another study recently revealed that AdMSCs mitigate oxidative stress and inflammation in the liver even after methotrexate-related damage [13]. However, the data in comparative studies between AdMSCs and BMSCs in liver fibrosis are very limited and, for long-term studies, are actually absent.

We, therefore, designed this study to compare the long-time persistence of favorable effects in male Wistar rats with liver fibrosis treated using MSCs derived from white adipose tissue (AdMSCs) and bone marrow (BMSCs). We aimed to determine whether one of these options is potentially preferrable or both are equally beneficial or not appropriate. To reach this aim, we modeled liver fibrosis with eleven subcutaneous injections of carbon tetrachloride solution for six weeks, followed by grafting AdMSCs or BMSCs to the fibrotic animals.

## 2. Results

### 2.1. Survival and Blood Tests: AdMSCs and BMSCs Show No Difference

**Figure 1:**
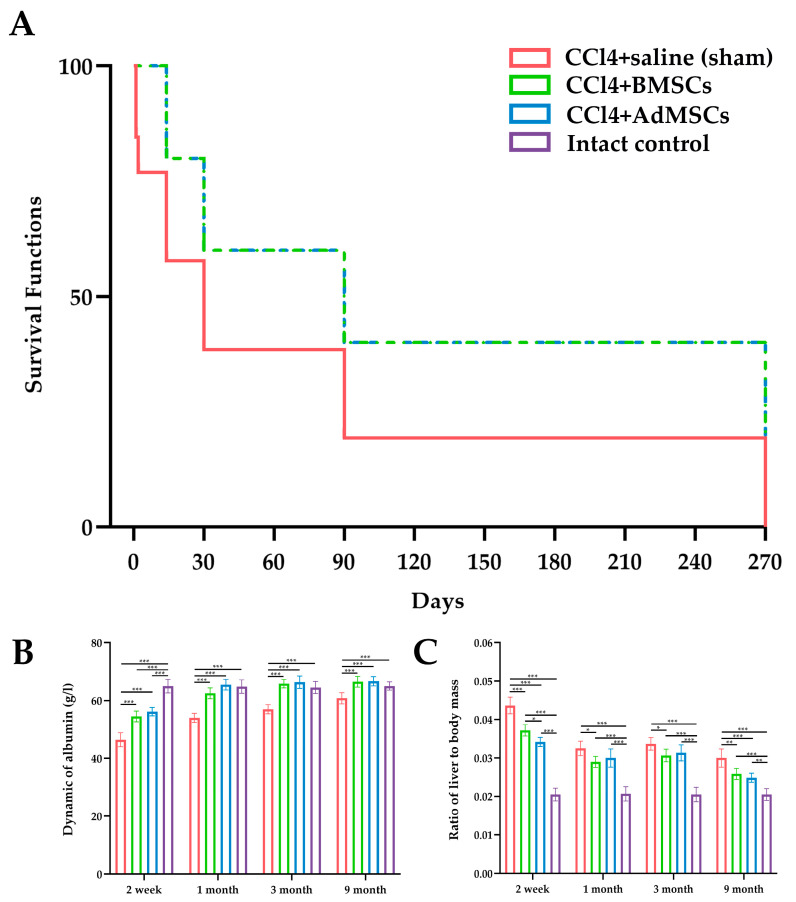
Animal survival and blood tests in liver fibrosis treated using either AdMSCs or BMSCs. (**A**) Kaplan–Meier plot of survival for groups receiving CCl_4_ poisoning. Lethality was increased in animals with sham treatment (CCl_4_ + saline), whereas other groups only experienced planned sacrifice at the study timepoints of Days 14, 30, 90, and 270. Analysis of variance with Tukey post hoc testing between the samples, * *p* ˂ 0.05, ** *p* ˂ 0.01, *** *p* ˂ 0.001: (**B**) Dynamics of blood albumin content; no difference between the MSC-treated groups found. (**C**) Liver-to-body mass ratio; the dynamics of the ratio are similar for both MSC approaches.

We assessed the survival rate between the study groups: treatment by BMSCs; treatment by AdMSCs; sham treatment (CCl_4_ + saline), vehicle control. Among the animals experiencing poisoning in the study, no extra mortality occurred in rats receiving MSCs on Day 7 after the last CCl_4_ injection (Figure 1A), whereas approximately one-fourth of saline-treated rats died due to liver pathology up to Day 14. However, from Day 14, we observed no additional lethality, and the number of animals staying alive accorded to the planned sacrifice at timepoints of Days 14, 30, 90, and 270 for blood tests and histological study.

**Figure 2:**
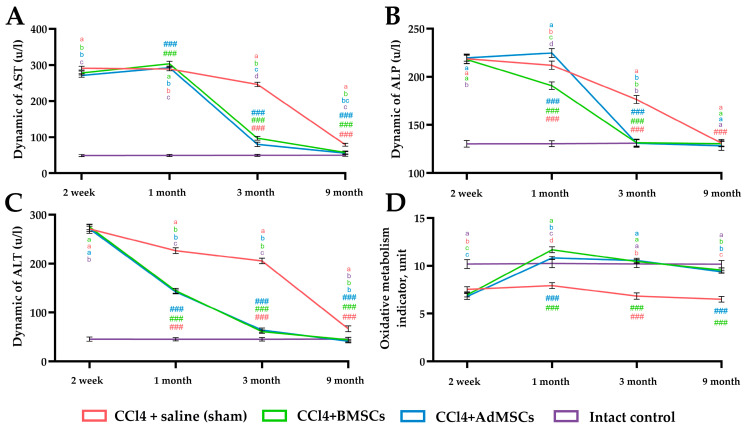
Blood tests in liver fibrosis treated using either AdMSCs or BMSCs. (**A**,**B**) Dynamics of aspartate and alanine aminotransferases (AST, ALT) in the blood; the values for both MSC-treated groups recover by Day 90 compared to animals with sham treatment (CCl_4_ + saline). (**C**) Dynamics of alkaline phosphatase (ALP) aminotransferase in the blood; the values for MSC-treated rats recover by Day 90 but with a lesser increase in the first month for the group of BMSCs. (**D**) Dynamics of oxidative index shows no difference between both approaches of MSC treatment; the values calculated with respect to activity rates of NADH and FAD as well as of blood flow oscillation amplitudes due to the myogenic, neurogenic, and cardiac regulation. Presentation of two-way ANOVA comparison: ### of corresponding colors shows difference from previous timepoint of the same group; letters mark the differences between corresponding group of corresponding color and: (a) sham treatment (CCl_4_ + saline); (b) CCl_4_ + BMSCs; (c) CCl_4_ + AdMSCs; (d) intact control.

Immediately before the sacrifice, animals were tested with a laser Doppler flowmetry device for quantification of the oxidation index in tail vessels (Figure 2D), tending to be higher in overall normal blood supply. As shown in the plot (Figure 2D), CCl_4_ poisoning with sham treatment was accompanied by decreased values of the oxidative index at all timepoints, with even a slight decline up to Day 270. At the same time, regimens of CCl_4_ with injections of AdMSCs and BMSCs resulted in a relatively rapid recovery of the oxidative index for the corresponding study groups. In both cases, this recovery occurred up to one month after the poisoning finished.

We also measured biochemical values of albumin, ALT, AST, and ALP in the venous blood of rats, whose results are given as plots (Figure 1B and Figure 2A–C). Albumin levels decreased in the first weeks for all groups with liver fibrosis, though they recovered almost completely for all experimental groups up to Day 270 (Figure 1B). Dynamics for the levels of aminotransferases (Figure 2A,B) stayed almost the same, except for a much more gradual recovery for the group with sham treatment. However, AdMSC- and BMSC-treated animals showed no differences between the samples in the values of aminotransferase content. Similarly, ALP levels did not vary between the MSC-treated groups (Figure 2C); BMSC-treated rats, nevertheless, demonstrated a more rapid recovery of ALP, already up to Day 30 after the end of CCl_4_ poisoning.

Having sacrificed the rats, we calculated the ratio between liver and body masses (Figure 1C). Initially elevated in all groups except the intact (control) animals, the ratio further decreased, almost recovering to the values of the control group in MSC-treated groups. However, the sham treatment CCl_4_-poisoned group had a 1.5-fold increase in the ratio, even up to Day 270.

### 2.2. Histological Study: A Slight Preference for AdMSCs

**Figure 3:**
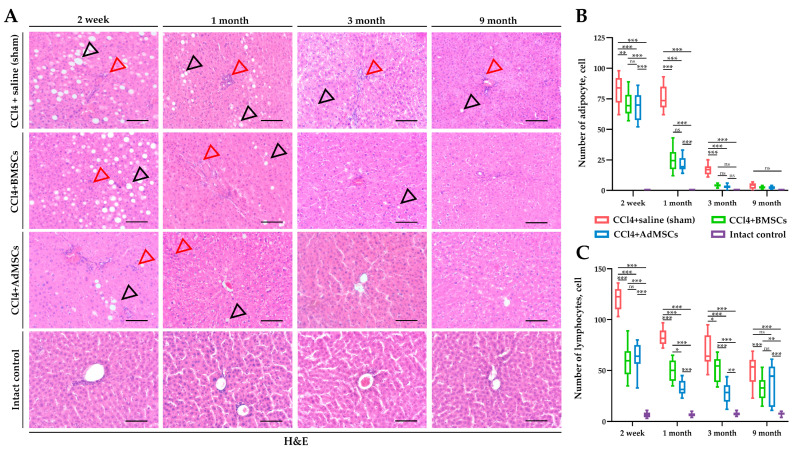
Liver fibrosis treated using AdMSCs and BMSCs. Hematoxylin-and-eosin staining. (**A**) Microphotographs of liver sections: red arrows show lymphocytes; black arrows show white adipocytes. Scale bar 100 μm. (**B**,**C**) Plots demonstrating the number of adipocytes (above) and lymphocytes (below). The difference between the marked groups (sham treatment, or saline, after CCl_4_; AdMSC- or BMSC-related treatment) is significant at: * *p* ˂ 0.05, ** *p* ˂ 0.01, *** *p* ˂ 0.001.

Figure 3 shows histological patterns in H&E staining. Two weeks after the poisoning, a clear image of dystrophic and inflammatory changes was seen in all the experimental groups. Interestingly, AdMSC-treated animals showed a significantly lower number of both adipocytes and lymphocytes at mid-term observation (up to first and third month). However, normal cell architecture recovered, both visually and according to the number of adipocytes (Figure 3B) and lymphocytes (Figure 3C) counted in all groups up to Day 270. Nonetheless, vehicle-treated animals after CCl_4_ still maintained some signs of dystrophy and lymphocytic infiltration on histological slides in long-term observation, though without significant differences.

**Figure 4:**
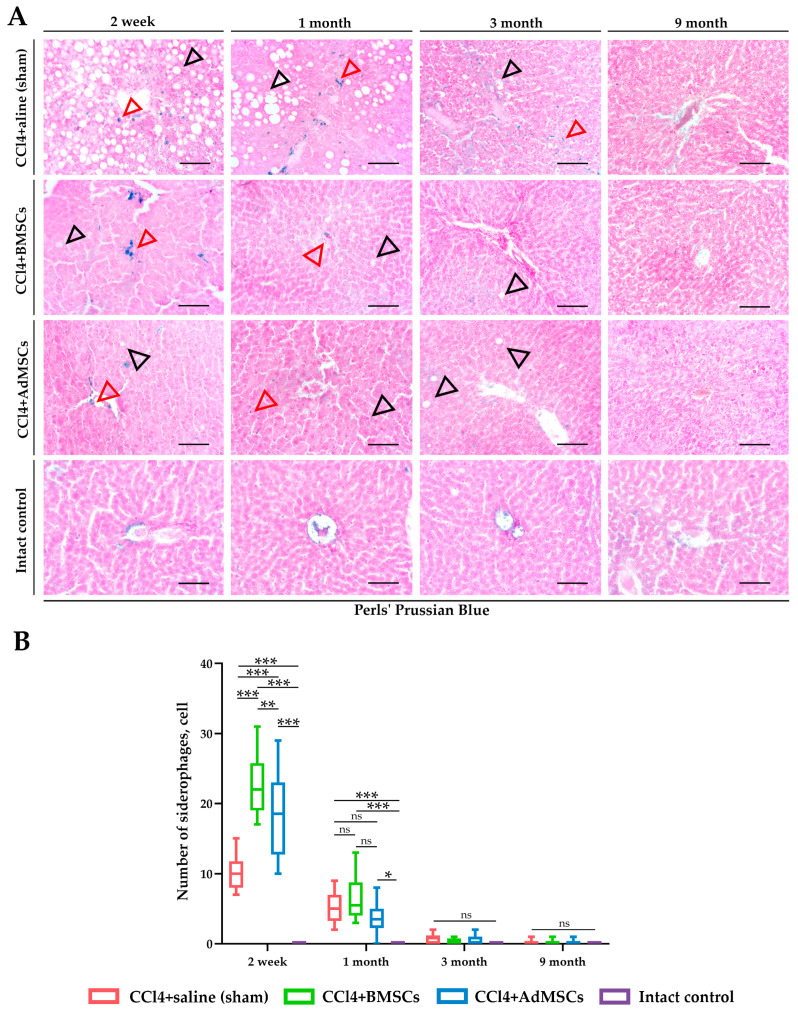
Liver fibrosis treated using AdMSCs and BMSCs. Perls Prussian blue staining. (**A**) Microphotographs of liver sections: red arrows show siderophages; black arrows show white adipocytes. Scale bar 100 μm. (**B**) Plot demonstrating the number of siderophages. The difference between the marked groups (sham treatment, or saline, after CCl_4_; AdMSC- or BMSC-related treatment) is significant at: * *p* ˂ 0.05, ** *p* ˂ 0.01, *** *p* ˂ 0.001.

Figure 4 demonstrates the siderophages (hemosiderin-accumulating macrophages) in the liver after Perls staining. Siderophages may appear due to the damage brought to hepatic plates and, therefore, to the blood flow through sinusoidal capillaries in hepatic lobules. Interestingly, in the first weeks, MSC-treated groups exhibited even higher hemosiderin deposits visually, together with dystrophic changes (Figure 4B). However, up to Day 270, only some solitary siderophages could be found in any group, without significant differences between the samples.

**Figure 5:**
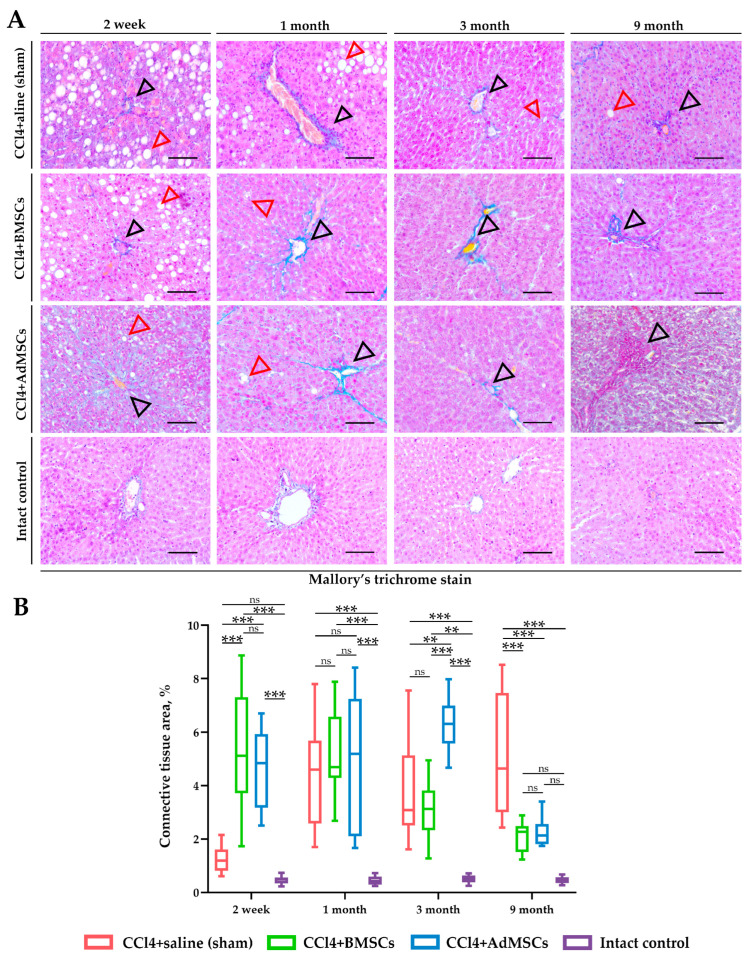
Liver fibrosis treated using AdMSCs and BMSCs. Mallory’s trichrome staining. (**A**) Microphotographs of liver sections: red arrows show white adipocytes; black arrows show the zones of fibrosis (blueish color). Scale bar 100 μm. (**B**) Plot demonstrating the area of connective tissue proper. The difference between the marked groups (sham treatment, or saline, after CCl_4_; AdMSC- or BMSC-related treatment) is significant at: ** *p* ˂ 0.01, *** *p* ˂ 0.001.

An even more interesting pattern of histological changes was observed with the assessment of relative area occupied by connective tissue proper after Mallory’s staining (Figure 5). Thus, MSC-treated groups experienced a large increase in collagen-containing areas (blue zones; marked by black arrows; Figure 5A). In addition, AdMSC-treated rats tended to develop significantly more prominent fibrosis for the first three months but, finally, both groups injected with AdMSCs and BMSCs showed more favorable values of connective tissue area compared to poisoned animals with sham treatment up to the end of the study (however, already without significant differences between the groups) (Day 270).

**Figure 6:**
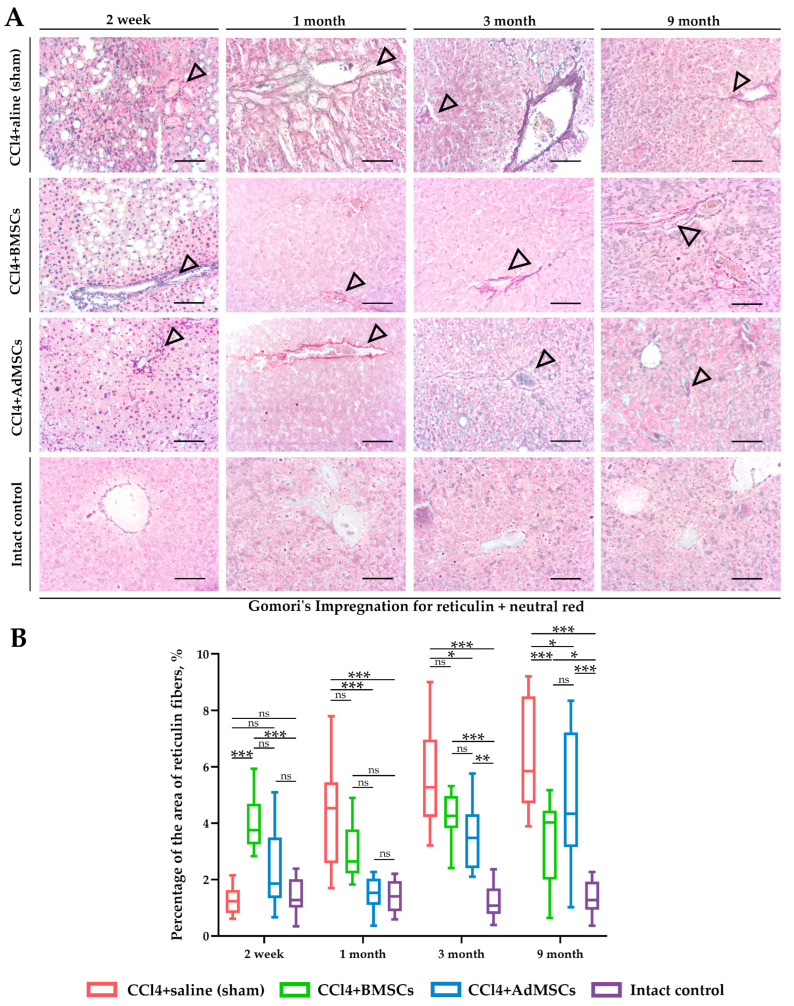
Liver fibrosis treated using AdMSCs and BMSCs. Gomori’s silver impregnation. (**A**) Microphotographs of liver sections: black arrows show the zones abundant with reticular fibers. Scale bar 100 μm. (**B**) Plot demonstrating the area occupied by reticular fibers (reticulin). The difference between the marked groups (sham treatment, or saline, after CCl_4_; AdMSC- or BMSC-related treatment) is significant at: * *p* ˂ 0.05, ** *p* ˂ 0.01, *** *p* ˂ 0.001.

In contrast, Gomori’s silver impregnation for the detection of reticulin fibers revealed a more profitable outcome in AdMSC-treated animals at the end of the study, though a visual one and not statistically confirmed (Figure 6). BMSC-treated rats exhibited a low extent of reticulin fibers (black arrows; Figure 6A), generally favorable for fibrosis regression, up to Day 270 (yet without significant difference from the AdMSC-treated group). Nevertheless, regarding the first month of observation (Figure 6B), the calculation demonstrated an even better rate of recovery in the BMSC-treated group (although, again, without statistical significance). This pattern did not persist for a long time, slightly seen by Day 90 and completely disappearing up to Day 270.

**Figure 7:**
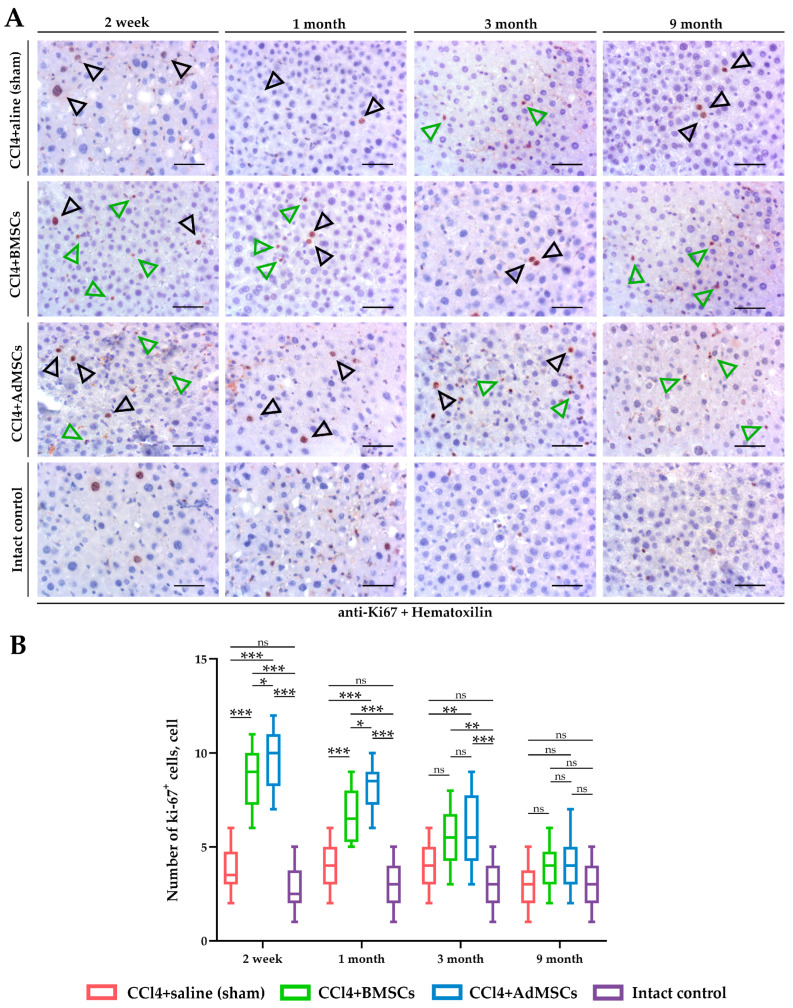
Liver fibrosis treated using AdMSCs and BMSCs. Anti-Ki67 staining. (**A**) Microphotographs of liver sections: black arrows show large Ki67-positive cells; green arrows mark smaller ones. Scale bar 100 μm. (**B**) Plot demonstrating the number of Ki67 cells per field of view. The difference between the marked groups (sham treatment, or saline, after CCl_4_; AdMSC- or BMSC-related treatment) is significant at: * *p* ˂ 0.05, ** *p* ˂ 0.01, *** *p* ˂ 0.001.

The immunohistochemical study with Ki67-positive (proliferating) cells also revealed a slight increase in their number at all timepoints for the AdMSC-treated group compared to others (Figure 7). However, this difference was only significant for the 1 month. The overall rate of proliferation tended to gradually decrease.

**Figure 8:**
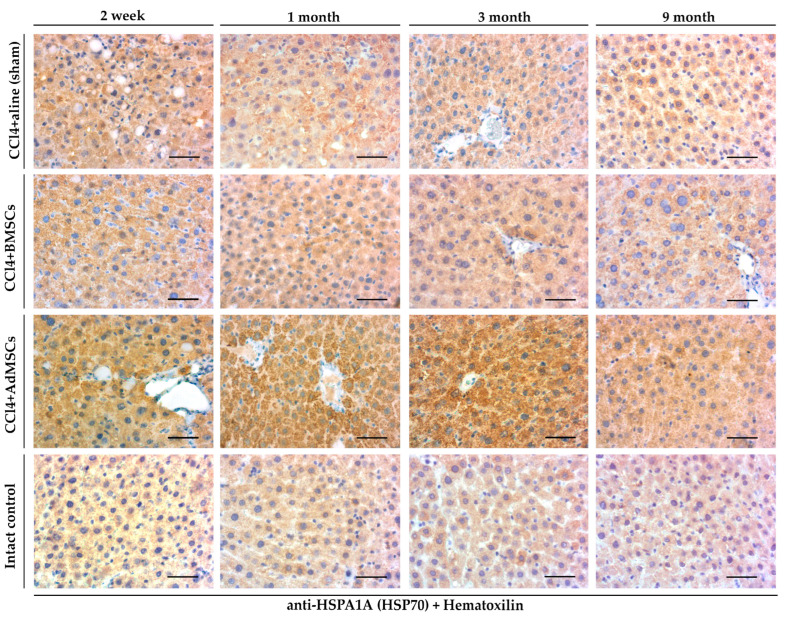
Liver fibrosis treated using AdMSCs and BMSCs. Anti-HSPA1A staining. Microphotographs of liver sections show HSPA1A expression (brown) in the cytoplasm of cells in liver parenchyma with barely seen expression in connective tissue areas. Scale bar 100 μm.

As the heat shock protein A1A from the HSP70 family, HSPA1A, is generally an inducible molecular chaperone, its expression may be a sign of a better intracellular reparation and proteome recovery. However, as one can see from Figure 8, HSPA1A is widely expressed in the cytoplasm of almost all cells in liver parenchyma at any timepoint without any visual difference; therefore, no reasonable quantification is available.

Therefore, the histological study demonstrates evidence of liver fibrosis development in all groups after poisoning with carbon tetrachloride, though with a different pattern of further dynamics between the groups receiving sham treatment, injections of AdMSCs, or injections of BMSCs in our long-term study up to Day 270.

## 3. Discussion

In this study, we provided long-term monitoring of the effects related to liver fibrosis treatment in rats using either AdMSCs or BMSCs. First of all, the survival rates differed between the groups. Indeed, liver fibrosis is highly lethal [14], so any proper liver fibrosis modeling inevitably prevents the survival of some animals and was, therefore, excluded from this study. This feature was shown for CCl_4_-induced fibrotic models in rats [15,16]. Therefore, the mortality in the group receiving sham treatment indicates the adequacy of the CCl_4_ model. Importantly, no mortality was found in animals receiving MSCs of any origin, proposing the efficacy of both MSC treatment modes.

Interestingly, for most tests, we revealed no significant differences between the groups with MSC injections at late timepoints (including the end of the study) considering all the blood markers (albumin, ALT, AST, ALP), liver mass-to-body mass ratio, and oxidative index calculated using laser Doppler flowmetry. Sharp changes in these parameters during the first month after the MSC grafting recovered almost completely by the three-month timepoint.

Nevertheless, considering the histological parameters, both MSC-treated groups exhibited the same rate of recovery in terms of the number of siderophages. Similarly, both MSC-treated groups showed an almost entire regression of fatty dystrophy measured by the number of adipocytes. In addition, we studied the expression of HSPA1A, an inducible molecular chaperone, without changes seen between the groups and, therefore, no evident sign of a different rate of proteome regeneration after differing modes of liver fibrosis treatment.

Still, a set of significant differences is important. At the midpoint of this study, the area of connective tissue proper remained slightly diminished in rats after BMSCs compared to those after AdMSCs. In addition, the group of AdMSC-treated rats had a minor yet significant increase in the number of lymphocytes compared to the BMSC-treated animals at the midpoint. These facts imply a beneficial role of BMSCs, though a weakly expressed one, compared to AdMSCs. However, these differences only existed at the midpoints, with no significant changes in the parameters up to Day 270.

AdMSCs also showed two favorable histological patterns. The area of reticulin fibers stayed larger in AdMSCs at the midpoints, yet without significance (but also, surprisingly, in CCl_4_-poisoned animals after sham treatment). Even more important, Ki67-positive cells were more numerous on the sections of livers from AdMSC-treated rats. Taking these facts together, we consider them to reveal a more preferrable potential of AdMSCs in our study. Briefly, early periods seem to be more representative of the benefit of BMSCs, although AdMSCs exhibit their gradual positive impact in mid-term and long-term observation.

The difference in terms of monitoring is probably crucial. A study with similar design, comparing the effects of BMSCs, AdMSCs, and liver-derived MSCs in Wistar rats with liver fibrosis, revealed less benefits for AdMSCs, with the last observational timepoint at 9 weeks [17]. However, that study used thioacetamide modelling of liver fibrosis and not a carbon tetrachloride-related one.

Our results are consistent with the general background of AdMSC studies in parenchymatous organs of abdominal cavities but represent new comparative data. Actually, successful AdMSC usage for liver damage treatment was first expressed in 2012 without a comparison to other MSC subtypes [18]. Even earlier, evidence revealing a better impact of AdMSCs over BMSCs appeared in 2011, but that study regarded the pancreas [19]. Studies in other pathologies, meanwhile, show a dual pattern of competition between AdMSCs and BMSCs. For instance, in murine sepsis, AdMSCs were less efficient, with anti-inflammatory IL-8 cytokine suppressed after the usage of AdMSCs [20].

The role revealed for AdMSCs in our study may be due to several mechanisms. First of all, AdMSCs were shown to affect the Salvador–Warts–Hippo pathway, thereby enhancing apoptosis and downregulating abnormal proliferation [21]. In addition, exosomes from AdMSCs, stimulated by hepatocyte growth factor, alleviated liver fibrosis via the PI3K/Akt/P38MAPK pathway [22]. Extracellular vesicles of AdMSCs also involved signaling via the Mas/Akt/Foxo1 axis, mitigating non-alcoholic fatty disease in mice [23].

Cytokine regulation is also involved in AdMSC-related regulation of liver damage. Thus, the recovery of initial IL-17 signaling was essential in AdMSC-mediated liver regeneration after ischemia–reperfusion injury, together with the tacrolimus [24]. Finally, inside hepatocytes, AdMSCs also affected the transcription of *Cyp7a1*, *Baat*, *Cyp27a1*, *Adh7*, *Slco1a4*, *Aldh1a1*, and *Adh7* genes, and, thereby, bile production and retinol metabolism [25].

Therefore, AdMSCs may be favorable for liver fibrosis treatment due to the multifaceted machinery [26]. Importantly, AdMSCs possess a lower level of “stemness” than BMSCs, thus implying fewer cancer-related risks.

However, some additional options exist to ameliorate AdMSC usage in liver fibrosis. Recent data showed hypoxic AdMSCs may provide a more prominent beneficial impact [27]. In addition, MSCs from lymphoid tissue of tonsils are perhaps more preferrable than AdMSCs, as shown in vitro. In vivo studies are required to understand the pattern of difference between their impacts [28].

### Study Limitations and Future Perspectives

The most interesting question raised by this study is on the ambiguous nature of the differences between the impact of AdMSCs and BMSCs at different timepoints. On the one hand, early-term observations unveil some beneficial roles of AdMSCs. On the other hand, long-term effects of AdMSCs are reported. We consider this discrepancy may arise not only from the properties of MSC subtypes or bias of standardization but also due to the different degrees of fibrosis in individual animals aggravating the already-challenging translation into human studies. We suppose that models in mammals with larger body and liver mass (e.g., primates) should be chosen for further testing of this combination of MSC subtype efficiency.

Additionally, study limitations include the evident potential to deepen the investigation of the machinery underlying the differences between the effects of AdMSCs and BMSCs in liver fibrosis with additional molecular methods, such as measuring the levels of proteins related to regenerative signaling with blotting assays (α-smooth muscle actin, collagen subtypes, pro- and anti-inflammatory cytokines), as well as total RNA and mRNA levels for these proteins with quantitative real-time PCR (and also for mRNA levels for liver enzymes such as ALT, ALT, ALP). Understanding more details of proteome recovery may also explain the nature of differences between the subtypes in our study. This relevant approach is common for studies of fibrosis machinery [29,30] and should be regarded further based on the findings of the current work. Additionally, future studies should assess the secretory profile of MSCs to estimate the impact of factors that reduce the accumulation of extracellular proteins. Animal studies should obviously be carefully extrapolated in future applications of clinical approaches, as there exist numerous biological and ethical restrictions.

## 4. Materials and Methods

### 4.1. Animal Care

Male Wistar rats (*n* = 180 in total) weighing 300 ± 2.5 g stayed in the animal facility of Shumakov National Medical Research Center of Transplantology and Artificial Organs, housed three per cage at a room temperature of 18–20 °C, with standard mixed food ad libitum and free access to water. The experimental interventions were provided in daytime in a temperature range of 22–24 °C.

### 4.2. Obtaining and Cultivating Stem Cells

We used thirty intact rats to obtain AdMSCs and BMSCs following the techniques we reported earlier [31,32] and in accordance with the consensus of the International Society for Cell & Gene Therapy [33]. Briefly, we performed liposuction from the medial inguinal region of the rats to obtain AdMSCs [34]. Lipoaspirate was removed in aseptic conditions into Hank’s balanced salt solution (Thermo Fisher Scientific, Waltham, MA, USA), enriched with penicillin, streptomycin, and amphotericin B (Gibco, Life Technologies, Carlsbad, CA, USA). After grinding and clostridiopeptidase A (Sigma-Aldrich, Burlington, MA, USA) processing, we centrifugated the suspension at 400 rpm for 10 min and then resuspended the sedimented cells for cultivation. Isolated cells were cultivated at 37 °C in 5% carbon dioxide for two passages in Dulbecco’s modified Eagle’s medium/F12 (PanEco, Gorki Leninskie, Russia) with 10% fetal bovine serum (HyClone, Logan, UT, USA) and N-2-hydroxyethylpiperazine-N-2-ethane sulfonic acid (Gibco, Carlsbad, CA, USA), entirely changing the cultivation medium every three days and filtering the cell culture through 100 μm pores (SPL Life Sciences, Pocheon, Republic of Korea) to remove the debris and unviable cells.

**Figure 9:**
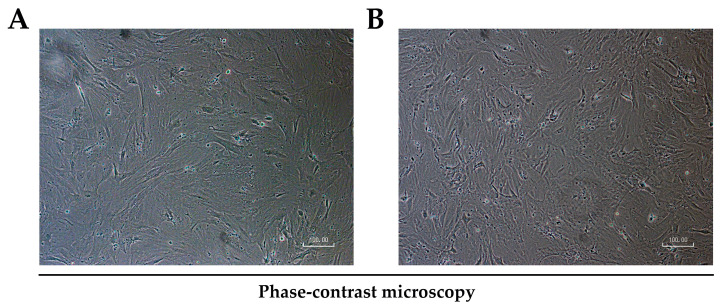
Cultivation of mesenchymal stem cells derived from adipose tissue (AdMSCs) or bone marrow (BMSCs) of Wistar rats. Phase microscopy of fibroblast-like cultivated stem cells after their 2nd passage: (**A**) AdMSCs, (**B**) BMSCs. Scale bar 100 μm.

The same manner of centrifugation and cultivation was relevant for bone marrow aspirate to obtain BMSCs, whereas cultivated cells of both origins, AdMSCs and BMSCs, can be observed in Figure 9. After the cultivation, we assessed the viability using phase-contrast microscopy for 3 min after 0.1% trypan blue staining. For cell-containing flasks with over 95% viability, we performed flow cytometry to only include cell cultures that are positive to CD73, CD90, CD105 and negative to CD11b, CD14, CD19, CD34, CD45, CD79a, and HLA-DR [33], for grafting. We used the cells suspended diluted at 1 × 10^6^/100 µL in phosphate-buffered saline as the dilution is common in studies [8].

### 4.3. Experimental Model and Study Design

**Figure 10:**
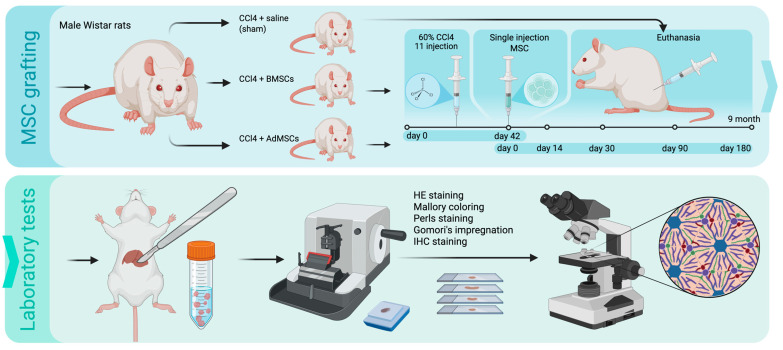
Study design: AdMSCs vs. BMSCs in chronic liver fibrosis. Obtained MSCs derived from adipose tissue (AdMSCs) or bone marrow (BMSCs) of healthy donor rats were further grafted to male Wistar rats that had received 42-day-long CCl_4_ poisoning. We performed laser Doppler flowmetry, blood tests (albumin, ALT, AST, ALP), and histological study to evaluate the dynamics of liver regeneration up to Day 270 after the end of poisoning. Created in BioRender. Kuzmin, E. (2026) https://BioRender.com/qlb7072.

The study design is shown in Figure 10. Thirty rats stayed intact as a control group. Other animals (*n* = 120) experienced eleven subcutaneous injections of 60% CCl_4_ solution in peach kernel oil over 42 days according to a previously approved dosage regimen [32,35,36]. In total, every animal received the CCl_4_ solution at a dose of 3.5 mL per 100 g of body mass. The choice of poisoning agent was based on the well-known hepatotoxicity induced by CCl_4_ [37,38,39,40].

Thirty animals died during the poisoning or in the first 7 days after it, and, therefore, these animals were excluded from the study. Ninety experimental rats that survived the CCl_4_ poisoning were randomly divided into three groups (*n* = 30 per group): treatment using BMSCs; treatment using AdMSCs; sham treatment (saline). These stem cell- and saline-treated animals received intraperitoneal injections on Day 7 after the end of poisoning; stem cells were given at a dose of 5 × 10^6^ cells (both AdMSCs and BMSCs) in 0.1 mL of saline.

At the timepoints of Days 14, 30, 90, and 270, we conducted laser Doppler flowmetry of hemodynamics in animals, which further received lethal intraperitoneal injections of body mass-related Zoletil-100 doses (Virbac, Carros, France). Immediately after the sacrifice, we performed blood sampling followed by autopsy and fixation of liver samples in 10% neutral-buffered formalin. We also weighed animals before the sacrifice and their livers after it to calculate liver-to-body mass ratio.

### 4.4. Laser Doppler Flowmetry

We employed a Lazma ST device (Lazma, Moscow, Russia) to study tissue microcirculation in the tails of rats, as proposed earlier [41]. The device permitted an automatized calculation of an index for oxidative metabolism from the values of registered parameters (activity rates of reduced nicotinamide adenine dinucleotide, NADH, and oxidized flavin adenine dinucleotide, FAD; blood flow oscillation amplitudes due to the myogenic, neurogenic, and cardiac regulation).

### 4.5. Biochemical Study

After the blood sampling, we conserved the serum in 3.8% sodium citrate solution after 10 min of centrifugation at 1500 rpm with Elmi CM-6M (Elmi, Riga, Latvia). Blood tests were performed with semi-automatic biochemical analyzer EMP-168VET (Chengdu Empsun Medical Co., Ltd., Chengdu, China) and reagents (Diavet, Serpukhov, Russia) for quantification of albumin, alanine aminotransferase (ALT), aspartate aminotransferase (AST), and alkaline phosphatase (ALP).

### 4.6. Histological Study

Liver tissue samples were fixed in 10% neutral-buffered formalin with further standard paraffinization and 3 μm sections obtained (*n* = 6 per each staining from any animal). We employed hematoxylin and eosin (H&E), Perls, Mallory’s, and Gomori’s staining as well as immunohistochemical study with anti-Ki67and anti-HSPA1A antibodies. Microphotographs were obtained with Axio Imager.A1 and Axiocam 305 in Zeiss Zen 3.10 (Zeiss, Oberkochen, Germany). Two measurements of blinded slides were provided by two laboratory workers to reduce inter- and intra-observer variability.

Cell and area calculations were performed with QuPath 0.7.0 [42]. We used microphotographs of H&E-stained slides to calculate the number of adipocytes and lymphocytes per field of view to evaluate the fatty dystrophy and inflammatory infiltration. Images of slides with Mallory’s staining allowed for the calculation of relative area with collagen fibers to evaluate fibrosis levels. Perls staining, which permits the detection of Fe^3+^ deposits in blood congestion upon fibrotic liver damage, was used to calculate the number of siderophages per field of view. Gomori’s staining (silver impregnation) revealed collagen type III fibers (reticular fibers), normal element of Disse spaces in the liver, to assess the level of reversible fibrotic changes (calculated as relative area). We also aimed to estimate indirect markers for the rate of proliferation and overall cell viability; for this, we preferred Ki67 as a common mitosis marker and HSP70 family of molecular chaperones as key players of proteostasis and viability [43].

Ki67-positive cells were calculated to assess the rate of proliferative regeneration in epithelial cells of liver parenchyma. We used 3 sections from each animal at any timepoint per group, with 6 areas analyzed per section, and included DAB-positive cells with 0.3 threshold of detection overpassed in the region of interest (equal to field of view size). HSPA1A was expressed in all cells and was maintained for qualitative analysis without quantitative assessment. Other fibrosis-related markers, such as α-smooth muscle actin, desmin, or transforming growth factor beta, were not tested as the model of fibrosis development has been successfully approved many times in our previous studies [35,36].

For immunohistochemical study, we employed anti-Ki67 (1:5000; clone SR00-02, article HA721115; Huabio, Hangzhou, China) and anti-HSPA1A (1:50; clone SA0379, article ET1601-11; Huabio, Hangzhou, China) monoclonal antibodies with 3,3′-diaminobenzidine as a chromogen. Sections were exposed to dewaxing/antigen retrieval with an appropriate buffer solution (E-IR-R220A; Elabscience, Wuhan, China), washed in phosphate-buffered saline and then placed into bovine serum albumin for 20 min at 37 °C. After incubation with primary antibodies (18 h at 37 °C) and washing with phosphate-buffered saline, we used secondary antibodies (2 h at 37 °C), conjugated with horseradish peroxidase (HA1119, M05-22-P2; Huabio, Hangzhou, China). Hematoxylin was used for counterstaining. Two paraffinized sections (skin and cartilage) served as positive and negative controls for both antibodies.

### 4.7. Statistical Study

We employed OriginPro 2024 (Origin Lab, Northampton, MA, USA) for calculations and Graph Prism 10.5.0 (Dotmatics, Boston, MA, USA) for plotting. The survival rate was calculated with Kaplan–Meier function. We estimated the distribution of the values for the other twelve parameters (oxidative index with laser Doppler flowmetry; albumin, ALT, AST, ALP levels; ratio of liver to body mass; the number of adipocytes, lymphocytes, siderophages, relative area of connective tissue proper, relative area of reticular fibers, the number of Ki67^+^ cells) with Shapiro–Wilk test, and we compared the samples with ANOVA and Tukey’s post hoc test for the Gaussian distribution or compared the median values with Kruskal–Wallis test and Dunn’s post hoc test for the non-Gaussian distribution. We regarded the difference between samples or median values to be significant at *p* ˂ 0.05.

## 5. Conclusions

In this study, we compared the long-time persistence of changes in the content of hepatic blood plasma markers and histological parameters in male Wistar rats with liver fibrosis treated using MSCs derived from white adipose tissue (AdMSCs) and bone marrow (BMSCs). Up to Day 270 of this study, both MSC-treated groups barely showed differences from animals with sham treatment in terms of the oxidative index and blood markers, although AdMSC-treated rats presented a more favorable histological pattern than BMSC-treated ones, considering the relative area of reticular fibers and Ki67 cell count. We consider that AdMSCs may be beneficial in liver fibrosis compared to BMSCs due to the better potential for liver tissue regeneration 9 months after the treatment with AdMSCs.

## Data Availability

The data presented in this study are available on reasonable request from the corresponding authors.

## Abbreviations

The following abbreviations are used in this manuscript:

AdMSCs	mesenchymal stem cells derived from white adipose tissue
BMSCs	bone marrow mesenchymal stromal stem cells
